# Design and Implementation of a Four-Unit Array Piezoelectric Bionic MEMS Vector Hydrophone

**DOI:** 10.3390/mi15040524

**Published:** 2024-04-14

**Authors:** Shuzheng Shi, Xiaoyong Zhang, Zhanying Wang, Liyong Ma, Kai Kang, Yongjun Pang, Hong Ma, Jinjiang Hu

**Affiliations:** 1 School of Mechanical Engineering, Hebei University of Architecture, Zhangjiakou 075000, China; shishuzheng2000@163.com (S.S.); wzy0313@126.com (Z.W.); maliyong@buaa.edu.cn (L.M.); kangkai_jgxy@163.com (K.K.); pyj1063@hebiace.edu.cn (Y.P.); 2 School of Computer Science and Engineering, North China University of Science and Technology, Tangshan 063210, China; 3 HBIS Group Co., Ltd., Shijiazhuang 050023, China; 4 Department of Intelligence and Automation, Taiyuan University, Taiyuan 030032, China; chn163@163.com; 5 School of Mechanical Engineering and Automation, Beihang University, Beijing 100191, China; 6 School of Materials Science and Engineering, University of Science and Technology Beijing, Beijing 100083, China

**Keywords:** PZT, MEMS, array vector hydrophone, bionic structure, sensitivity

## Abstract

High-performance vector hydrophones have been gaining attention for underwater target-monitoring applications. Nevertheless, there exists the mutual constraint between sensitivity and bandwidth of a single hydrophone. To solve this problem, a four-unit array piezoelectric bionic MEMS vector hydrophone (FPVH) was developed in this paper, which has a cross-beam and a bionic fish-lateral-line-nerve-cell-cilia unit array structure. Simulation analysis and optimization in the design of the bionic microstructure have been performed by COMSOL 6.1 software to determine the structure dimensions and the lead zirconate titanate (PZT) thin film distribution. The FPVH was manufactured using MEMS technology and tested in a standing wave bucket. The results indicate that the FPVH has a sensitivity of up to −167.93 dB@1000 Hz (0 dB = 1 V/μPa), which is 12 dB higher than that of the one-unit piezoelectric MEMS vector hydrophone (OPVH). Additionally, the working bandwidth of the FPVH reaches 20 Hz~1200 Hz, exhibiting a good cosine curve with an 8-shape. This work paves a new way for the development of multi-unit piezoelectric vector hydrophones for underwater acoustic detectors.

## 1. Introduction

Biomimicry [1,2], as a method of studying biological systems, plays an essential role in the development of novel sensors. The sensory organs of living organisms are diverse, ranging from the touch of invertebrates to the hairs and skin of vertebrates to the hair-like sensory organs of flying insects to the lateral lines of marine organisms, and even to the sharp and precise perception of the human cochlea [3,4]. Bionic hearing can significantly improve the performance and application potential of artificial hearing systems, allowing robots to operate more freely in the deep sea. Micro-electro-mechanical system (MEMS) sensors are the core of the bionic hearing system [5,6]. The bionics principle can be used to design bionic MEMS hydrophones for detecting underwater targets. At present, most of the MEMS vector hydrophones studied by scholars are single units with a bionic cilia fabricated on a cross-beam structure. In 2007, Xue et al. [7] proposed a one-unit cilia-MEMS vector hydrophone (OCVH) with a sensitivity of −197.7 dB@1 kHz (0 dB = 1 V/μPa) using bionic- fish-lateral-line cilia. As a component of the OCVH, the bionic cilia are able to pick up sound signals from underwater. In 2017, Bai et al. [8] reported a cross-supporting planar MEMS piezoresistive hydrophone with a sensitivity of about −210.2 dB@300 Hz, which was inspired by the tactile cells of seal whiskers. In 2020, our research group [9] reported on an MEMS piezoelectric vector hydrophone (OPVH) with four cantilever beams based on bionic cilia. The OPVH has a receiving sensitivity of −189.3 dB@920 Hz and a good low-frequency response at 20~1200 Hz. Bionic MEMS vector hydrophones provide a superior low-frequency response through optimization of their structure and bionic cilia. However, the higher sensitivity often comes at the expense of reduced working bandwidth.

To overcome the contradiction between hydrophone sensitivity and bandwidth, scholars are exploring the use of an array structure as a solution [10]. Based on the microcolumn cilia, Liu et al. [11,12] reported two kinds of array piezoresistive MEMS vector hydrophones. The first hydrophone is an array hydrophone consisting of four cross-beam units with different lengths of cilia and a highest sensitivity up to −189 dB at 20~5000 Hz. The second one is a multi-unit array hydrophone that achieved excellent consistency across its four units and a receiver sensitivity of −194 dB within the frequency range of 20 to 1000 Hz. Zhang et al. [13] proposed a four-unit piezoresistive vector hydrophone (FCVH) for which the sensitivity of FCVH is −188.5 dB, which is 11.8 dB higher than that for OCVH. These studies showed that an array structure has great potential to improve the sensitivity of the MEMS piezoresistive vector hydrophone while maintaining a satisfactory bandwidth. However, the use of varistors as sensitive units results in high temperature sensitivity, poor anti-interference, and a narrow dynamic response range. In addition, the piezoresistive hydrophones are highly power-dependent and require an external power supply. Piezoelectric sensors have garnered significant interest due to their excellent anti-noise capabilities and low power consumption. Their simple resonant structure allows for both excitation and sensing functions. In particular, piezoelectric array sensors can produce a large voltage output with a small displacement input, resulting in high sensitivity over a wide bandwidth [14]. In 2016, Xu et al. [15] developed a 5 × 5 array piezoelectric MEMS hydrophone at AIN-on-SOI platform, which demonstrated a sensitivity of −182.5 dB in the low-frequency range of 10~100 Hz. Based on the aluminum nitride (AlN) piezoelectric crystal, Wu et al. [16] reported an 8 × 9 array MEMS hydrophone with an array with a honeycomb structure, which achieved an acoustic pressure sensitivity of −178 dB over a bandwidth of 10 Hz~50 kH. Then, when they doped 9.5% scandium into the AlN, the receiving sensitivity of scandium-doped hydrophone went up to −164.5 dB at 10 Hz~50 kHz [17]. Although these studies have improved the sensitivity and bandwidth of the hydrophone through an array structure and material replacement, there is still a lack of systematic studies on the directional detection function. The bionic cilia structure can be applied to MEMS hydrophones as a sensitive receiving unit, while a piezoelectric transducer array structure can effectively improve the sensitivity and bandwidth, with good directional detection function [18]. The organic combination of the two structures not only meets the sensor requirements of sensitive reception to underwater acoustic signals, but also satisfies the passive testing characteristics, providing a novel bionic method for the design and implementation of piezoelectric MEMS vector hydrophones.

Based on our previous research and the advantages of the array structure, an FPVH was designed and fabricated with a bio-inspired fish-cilia-array structure. The structure parameters were simulated and optimized through finite element simulation and the sensor was manufactured using MEMS technology. The array structure was employed to solve the trade-off between the bandwidth and sensitivity of the hydrophone to a certain extent. The sensitivity and directionality of the FPVH was tested by comparative calibration experiment, which proves that the FPVH has high performance and research prospects.

## 2. Materials and Methods Sensor Principle and Structural Design

### 2.1. Bionic Principle

**Figure 1:**
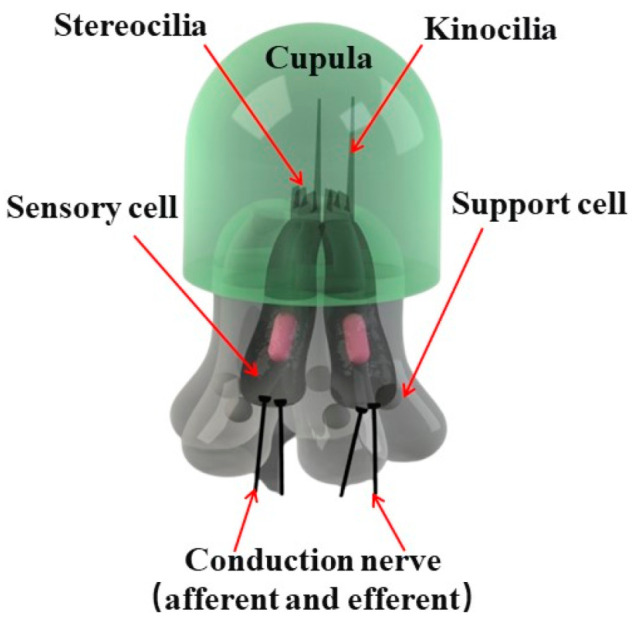
The bionic schematic of a fish neuromast.

Fish and aquatic amphibians have unique lateral line organs on both sides of their bodies that can sense temperature, pressure, and low-frequency vibration in liquid environments [19]. As shown in Figure 1, the neuromast within the lateral line organ is composed of multiple sensory cells with a long kinocilium and a few short stereocilia. The cupula is a glial secretion that condenses on the outer surface of the sensory organ and wraps around the kinocilia and stereocilia. The sensory pathway of the lateral line organ begins with pressure in the liquid environment entering the lateral tube through the lateral line hole, causing mucus to flow toward the neuromast and resulting in the movement of cupula. The mucus flow of the cupula drives the deflection of the kinocilia, and the hair cells are stimulated at that moment.

### 2.2. Structure of the Hydrophone

**Figure 2:**
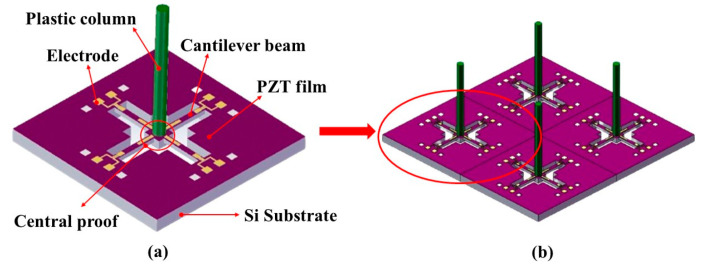
Bionic structural model of (**a**) OPVH and (**b**) FPVH.

The bionic structural design of the OPVH is expressed in Figure 2a. The OPVH is composed of four main components: the cross-beam with symmetrical cantilever beams, the PZT piezoelectric film, the columnar tactile cilium, and the central inertial unit. The most notable design aspect for the bionic sensor is the use of piezoelectric films to simulate the piezoelectric transducer unit of a sensory cell, while the acoustic cylinder is used to simulate the tactile cilia of the acoustic receiver signal. Based on this OPVH structure, the FPVH has been developed as consisting of four cross-beam cilium units as shown in Figure 2b. The PZT film covers the entire beam as the functional material, and the electrode is grown on the film. The layout of the piezoelectric element is determined by the size and distribution of the electrode. The bionic four-cross-beam-cilium array structure is used to detect underwater acoustic information based on the above bionic principle.

According to the acoustic receiving theory [7], when the hydrophone is moving freely underwater with *ka* is much less than 1 (*k* and *a* represent the wavenumber and the maximum geometric size of the vibrating pickup unit, respectively), there is no discernible distortion in the acoustic field surrounding the vibrating unit. The vibrational velocity of the pickup vibration unit (*V_s_*) can be described as follows:

(1) $$V_{s}=\frac{3\rho_{0}}{2\rho_{s}+\rho_{0}}V_{0}$$

where the vibrational velocity of a water particle is denoted as *V*_0_. The density of water and the bionic cilia are denoted as *ρ*_0_ and *ρ_s_*, respectively. Therefore, when *ρ_s_* is approximately equal to *ρ*_0_, the hydrophone can drive the pickup vibration unit to vibrate synchronously.

The vibrational information of water particles can be expressed through the vibrating amplitude and phases of bionic cilia. The density of polyethylene (PE) is similar to that of water, with a density of 0.95 × 10^3^ kg·m^−3^. In this study, four ciliated cylinders were fabricated using PE, due to its toughness and rigidity, which allows for the easy transmission of external stress. The cylindrical cilia transmit the signal, driving the central mass unit to rotate and the cantilever beam to vibrate. Opposing charges are produced on the top and bottom surfaces of the piezoelectric thin film due to beam deformation. The cantilever beam has piezoelectric lead zirconate titanate (PZT) films that detect underwater acoustic vector signals through their output voltage. Only the performance of the OPVH will be discussed as the individual OPVHs of the FPVH are identical.

### 2.3. Stress and Resonance Frequencies of the Cantilever Beam

**Figure 3:**
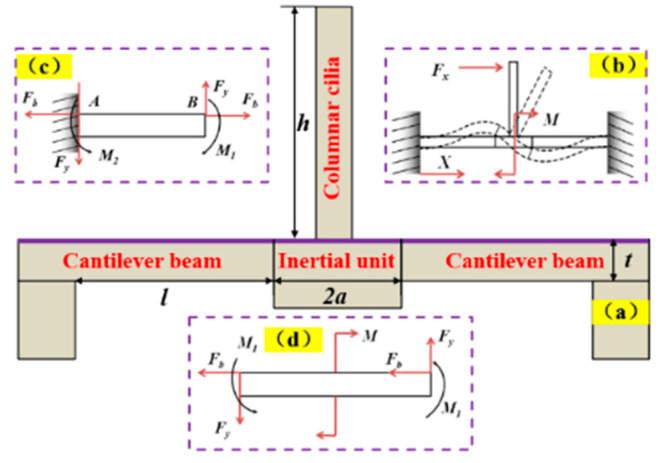
The cross-sectional structure and mechanical model of the microstructure. (**a**) Schematic of the sectional geometry of the cantilever beam microstructure, (**b**) sensitive structure section subjected to bending moment *M*, (**c**) force and moment of single cantilever beam, and (**d**) force analysis of the central inertial unit.

According to the relevant elastic mechanics and material mechanics [20,21], the cross-sectional structure and mechanical model of the microstructure were established as shown in Figure 3. Both the stress and the resonance frequency of the microstructure are analyzed while receiving a vibrating signal. The piezoelectric variation on the beam and the hydrophone sensitivity are affected by stress, while the working bandwidth is affected by the resonance frequency. During the analysis, the three assumptions should be made: (1) the cilia and the central inertial unit are rigid, and their deformation can be ignored; (2) the cilia and the central inertial unit are rigidly connected; and (3) the influence of damping is not considered. The microstructure section model was created as shown in Figure 3a. The cantilever beam dimensions are length *l*, width *b*, and thickness *t*. The *h* denotes the length of columnar cilia, and the central inertial unit has a side length of 2*a*. A flexural mechanical model of microstructure is constructed to analyze its stress and frequency characteristics. The deformation of the sensitive structure section under the bending moment *M* is shown in Figure 3b. A force of *F_x_* was applied to the columnar cilia, resulting in two separate components in the X and Y directions. The force and moment of a single cantilever beam are shown in Figure 3c. The horizontal component acting on the central inertial connection along the X-axis is analyzed as shown in Figure 3d. The other part rotates the moment *M*_1_ around the Y-axis at the center of the central inertial link. According to the moment–balance relationship, the moment *M*(*x*) of the beam at any point can be represented by [20]:

(2) $$M_{\left(x\right)}=-M_{1}+F_{y}(l-x)$$

where *M*_1_ denotes the moment at point B, *F_y_* denotes the vertical force on point B, and *x* denotes the distance from any point to the midpoint of the beam. The rotation angle *θ*_(x)_ can be calculated using from Equation (3),

(3) $$\theta_{\left(x\right)}=\int_{0}^{x}\frac{M_{\left(x\right)}}{EI}dx=\frac{1}{EI}[(-M_{1}+F_{y}l)x-\frac{1}{2}F_{y}x^{2}]$$

where *E* denotes the Young’s modulus of beam, *I* denotes the moment of inertia. According to Equation (3), the deflection *v_x_* can be expressed as follows:

(4) $$v_{x}=\int_{0}^{x}\theta_{\left(x\right)}dx=\frac{1}{EI}[\frac{1}{2}(-M_{1}+F_{y}l)x^{2}-\frac{1}{6}F_{y}x^{3}]$$

According to the continuity condition, the vertical displacement of the edge of the central inertial unit should be equal to the deflection at the end of the cantilever beam,

(5) $$v_{\left(l\right)}=a(-\theta_{\left(l\right)})$$

where *a* denotes the half-side length of the central inertial unit. By combining Equations (3)–(5), the following Equation (6) can be obtained

(6) $$\frac{1}{EI}[\frac{1}{2}(-M_{1}+F_{y}l)l^{2}-\frac{1}{6}F_{y}l^{3}]=a\left\{-\frac{1}{EI}[(-M_{1}+F_{y}l)x-\frac{1}{2}F_{y}x^{2}]\right\}$$

The vertical force *F_y_* on point B is given by the following:

(7) $$F_{y}=\frac{3(l+2a)}{l(2l+3a)}M_{1}$$

As shown in Figure 3d, according to the torque–balance relationship ∑*M* = 0, the following Equation (8) can be obtained

(8) $$2(F_{y}\cdot a+M_{1})=M$$

By substituting Equation (7) into Equation (8) can be expressed as follows:

(9) $$M_{1}=\frac{l(2l+3a)}{4(l^{2}+3al+3a^{2})}M$$

The moment of any point *x* is expressed as follows:

(10) $$M_{\left(x\right)}=\frac{l^{2}+3al-3x(a+l)}{4(l^{2}+3al+3a^{2})}M$$

Under the bending moment *M*_(*x*)_, the stress *σ*_(*x*)_ of *x* on the corresponding cantilever beam is expressed as follows:

(11) $$\sigma_{\left(x\right)}=\frac{M_{\left(x\right)}}{W}=\frac{l^{2}+3al-3x(a+l)}{4W(l^{2}+3al+3a^{2})}M$$

where the bending section modulus *W* can be expressed as

(12) $$W=\frac{bt^{2}}{6}$$

By substituting Equations (10) and (12) into Equation (11), we can get Equation (13).

(13) $$\sigma_{\left(x\right)}=\frac{M_{\left(x\right)}}{W}=\frac{l^{2}+3al-3x(a+l)}{\frac{2}{3}bt^{2}(l^{2}+3al+3a^{2})}M$$

Under the action of horizontal force *F_b_*, the elongation index of beam λ is expressed as follows:

(14) $$\lambda =\frac{F_{b}l}{Ebt}$$

Combined with *F_b_* and *M*_(*x*)_, the stress *σ*_(*x*)_ is expressed as follows:

(15) $$\sigma_{\left(x\right)}=\frac{l^{2}+3al-3x(a+l)}{\frac{2}{3}bt^{2}(l^{2}+3al+3a^{2})}F_{x}h+\frac{F_{H}}{bt}$$

According to the above analysis, the stress is proportional to the dimensions *l* and *h*, while it is inversely proportional to *b*, *t*, and *a*. Based on the piezoelectric effect, the sensing performance of the microstructure is determined by the PZT thin film. The charge density of the piezoelectric unit is proportional to the magnitude of the stress. The greater the change in charge density, the higher the output voltage. Therefore, the sensitivity increases as the stress on the beam increases. The sensitivity of the hydrophone is directly proportional to the maximum stress on the cantilever beam. To increase the output voltage, the piezoelectric sensor is positioned where stress concentration occurs.

In addition, working bandwidth is also a crucial parameter of the hydrophone. Based on the above analysis of the stress state of the microstructure, it can be seen that the vertical upward force *F_y_* and the moment *M* act together on the connection between the central inertial body and the cantilever beam. The displacement at point *A*, on the connection edge between the cantilever beam and the base is *d_i_*, and the angle between the cantilever beam and the X-axis is *θ_i_*. Figure 3b shows the stress and moment state of the cantilever beam. According to the moment–balance relationship, the moment *M*_1_(x) can be expressed as [21]:

(16) $$\frac{1}{2}F_{x}h=M_{1}+F_{y}a$$

The stiffness coefficients *K*_1_, *K*_2_, *K*_3_, and *K*_4_ corresponding to the displacement and rotation changes generated on the cantilever beam are expressed as follows:

(17) $$\{\begin{array}{cc} K_{1}=\frac{12EI_{y}}{l^{3}}=\frac{Ebt^{3}}{l^{3}} & K_{2}=\frac{6EI_{y}}{l^{3}}=\frac{Ebt^{3}}{2l^{2}}\\ K_{3}=\frac{3EI_{y}}{l^{3}}=\frac{Ebt^{3}}{3l^{2}} & K_{4}=\frac{6EI_{y}}{l^{3}}=\frac{Ebt^{3}}{2l^{2}} \end{array}$$

Combined with Equation (16) and Equation (17), this can be expressed as

(18) $$\{\begin{array}{c} \frac{1}{2}F_{x}h=K_{1}d_{i}w_{i}+K_{2}d_{i}+K_{3}\theta_{i}+K_{4}\theta_{i}w_{i}\\ d_{i}=\theta_{i}w_{i}\\ K_{2}=K_{4} \end{array}$$

By simplifying Equation (18), this can be expressed as

(19) $$F_{x}=2\frac{Ebt^{3}}{lh^{2}}(\frac{a^{2}}{l^{2}}+\frac{a}{l}+\frac{1}{3})d_{s}$$

The stiffness *K_x_* or *K_y_* of the cantilever beam in the X- or Y-axis is expressed as

(20) $$K_{x}=K_{y}=\frac{F_{x}}{d_{s}}=2\frac{Ebt^{3}}{lh^{2}}(\frac{a^{2}}{l^{2}}+\frac{a}{l}+\frac{1}{3})$$

The resonance frequency of the microdevice is expressed as follows

(21) $$f=\frac{1}{2\pi }\sqrt{\frac{K}{m}}=\frac{1}{2\pi }\sqrt{2\frac{Ebt^{3}}{mlh^{2}}(\frac{a^{2}}{l^{2}}+\frac{a}{l}+\frac{1}{3})}$$

According to the above analysis, the resonance frequency is proportional to the dimensions *b*, *t*, and *a*, while it is inversely proportional to *l* and *h*. The stress and resonant frequency vary inversely. Theoretically, increasing the maximum load on the cantilever beam will increase the sensitivity of hydrophone. However, this will reduce the resonance frequency, which in turn reduces the working bandwidth. The sensitivity and bandwidth are interdependent. Achieving both high sensitivity and a wide working range in a hydrophone can be accomplished through implementing an array microstructure, which is an efficient method for improving its limitations [12].

## 3. Sensitivity Gains of Multi-Unit Hydrophones

A multi-unit vector hydrophone outputs two voltage signals in each unit, both of which are in the horizontal plane. The X-channel and Y-channel outputs have identical characteristics, as they measure two mutually perpendicular components of the hydrophone. Taking the X-channel output of each array unit as an example, the X-channel outputs of an *N*-unit vector hydrophone are denoted as *U*_1_(t), *U*_2_(t)…*U_n_*(t), respectively. The output voltage of each array unit is given by Equation (22) [13].

(22) $$\{\begin{array}{c} U_{1}\left(t\right)=u_{1}\left(t\right)\\ U_{2}\left(t\right)=u_{2}\left(t\right)\\ ...\\ U_{N}\left(t\right)=u_{N}\left(t\right) \end{array}$$

where *u*_1_(t), *u*_2_(t)…*u_N_*(t) are the response of each array unit to the target acoustic signal, respectively. The linear summing function of each output signal is achieved by an adder formed by an operational amplifier circuit. The average power meter of the output of each array unit in response to the target acoustic signal can be expressed as follows:

(23) $$\bar{U^{2}}=\bar{{\left[u_{1}\left(t\right)+u_{2}\left(t\right)+\cdot \cdot \cdot +u_{N}\left(t\right)\right]}^{2}}$$

Expansion of Equation (23) yields the following:

(24) $$\bar{U^{2}}=\bar{u_{1}\left(t\right)u_{1}\left(t\right)+\cdot \cdot \cdot u_{N}\left(t\right)u_{1}\left(t\right)+\cdot \cdot \cdot u_{1}\left(t\right)u_{N}\left(t\right)+\cdot \cdot \cdot u_{N}\left(t\right)u_{N}\left(t\right)}$$

When each unit in the array has equal sensitivity, they will produce the same amplitude response to the target signal, resulting in an output signal with consistent average power $\bar{u^{2}}$. Equation (24) can be simplified as follows:

(25) $$\bar{U^{2}}=\bar{u^{2}}\sum_{j}{\sum_{i}\left(r_{s}\right)}_{ij}$$

where (*r_s_*)*_ij_* represents the number of interrelationships between the signals in the output of the *i*-th and the *j*-th array units. In a multi-sensor unit vector hydrophone with an *N* × *N* array, the two array units at the diagonal ends of the entire sensitive unit are farthest apart, with a spacing expressed as *a* = 2.4(*N* − 1) mm. Therefore, the maximum value of the number of interrelationships among the output signals of each array unit can be determined by the input signal frequency and this spacing. The number of interrelationships can be expressed as shown in Equation (26).

(26) $${\left(r_{s}\right)}_{ij}=\cos\left[2\pi \frac{2.4\left(N-1\right)f}{c\times 10^{3}}\right]$$

where *f* is the input acoustic signal frequency, *N* is the number of arrays and *c* is the acoustic velocity in the water.

**Figure 4:**
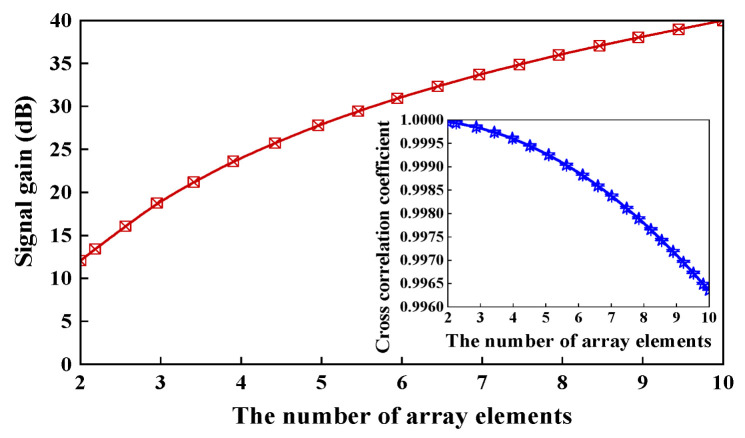
Signal gain when the number of array elements changes and cross correlation coefficient.

To visualize the impact of phase differences between the array units on the number of interrelationships, we calculated the interrelationship number using an input acoustic signal frequency of 1000 Hz and a sound speed of 1500 m/s in water. The number of array units was increased from 4 (2 × 2) to 100 (10 × 10), and the interrelationship number of the two farthest array elements on the diagonal of the multi-sensor unit hydrophone was calculated, as illustrated in Figure 4. As the number of elements increases, the number of interrelationships decreases to 0.9959. Specifically, when the number of elements is 4, the maximum phase difference is 0.58° and the number of interrelationships is 0.999. It can be seen that the number of interrelationships between the units has little effect when the number of elements is small. The sensitivity gain of the multiple hydrophones can be obtained by the signal gain compared to a single unit is expressed as follows

(27) $$G=10\mathrm{lg}\frac{\bar{U^{2}}}{\bar{u^{2}}}=10\mathrm{lg}\sum_{j}\sum_{i}{\left(r_{s}\right)}_{ij}$$

By substituting the interrelationships value (*r_s_*)*_ij_* into Equation (28), the signal gain of the multi-unit hydrophone can be obtained from 4 to 100. When the number is 4, the gain is 12.039 dB, which is very close to the theoretical value of 12.041 dB. It can be seen in the inset of Figure 4 that (*r_s_*)_2×2_ ≈ 1. By increasing the number of units to 100, the gain will be increased to 39.991 dB, which is also close to 40 dB. Therefore, the spacing of the units has very little effect on the sensitivity gain. With fewer units, the gain in sensitivity of the multi-unit hydrophone is close to the ideal value of 20lg*N*.

## 4. Directional Principle of MEMS Vector Hydrophone

Based on the principle of underwater acoustics [22], a sound source emits a signal that disturbs water particles, causing the system to become unbalanced. This results in a force that returns the system to its original equilibrium state, allowing for the propagation of acoustic signals in a liquid environment. Mechanical disturbances facilitate the emergence of various forms of state information in the water medium, including the sound field. The sound field is the region where sound waves exist. In an ideal fluid medium, the Euler equation [23] is used to describe the nonlinear relationship between the state vibration velocity *v*(*r*,*t*) and the sound pressure *p*(*r*,*t*) at any point in the sound field,

(28) $$\{\begin{array}{c} \frac{\partial v\left(r,t\right)}{\partial t}+\frac{1}{\rho }\nabla p=0\\ v\left(r,t\right)=-\frac{1}{\rho }\int \nabla pdt \end{array}$$

where ▽*p* is the sound-pressure gradient and *ρ* is the density of the liquid medium.

**Figure 5:**
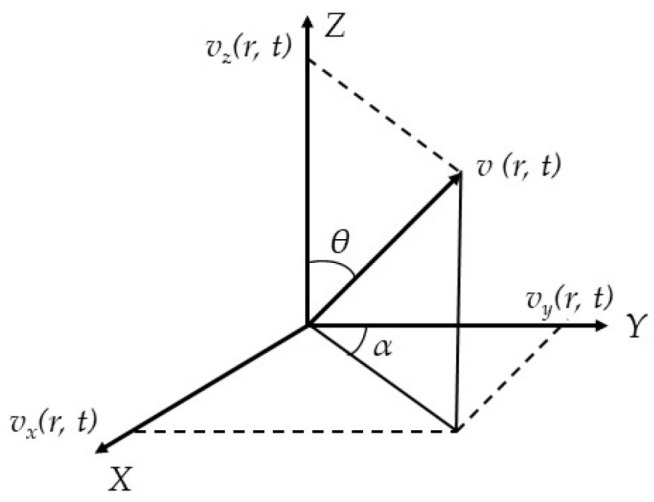
Orthogonal decomposition of vibration velocity.

In the continuum field, the orthogonal decomposition model of the vibration velocity projection in the Cartesian coordinate system is established, as shown in Figure 5. When the vector sensor satisfies the condition of picking up vibration, the propagation of sound waves is determined by the medium in an infinite-ideal-uniform sound field in the same direction. The medium density, vibration velocity and pressure of the acoustic wave are denoted as *ρ*(*r*,*t*), *v*(*r*,*t*), and *p*(*r*,*t*), respectively.

The sound pressure of the underwater acoustic plane wave is expressed as the superposition of simple harmonic plane waves,

(29) $$p(r,t)=\int X(\omega )e^{j(\omega t-kr)}d\omega$$

(30) $$X(\omega )=\int x(t)e^{-j\omega t}dt$$

where *x*(*t*) is the sound-pressure wave, *X(ω)* is the frequency spectrum, and $r=\sqrt{x^{2}+y^{2}+z^{2}}$ is the distance between the point and the sound source.

Using simultaneous Equations (28)–(30), the vibration velocity can be obtained as follows:

(31) $$v\left(r,t\right)=\frac{1}{\rho c}\left[\cos\theta \cos a\cdot \xi +\sin\theta \cos a\cdot \eta +\sin a\cdot \zeta \right]p(r,t)$$

where *θ* is the angle between the horizontal projection vector of the wave and the X-axis, *θ*∈[0, 2π], *α* is the angle between the wave vector and the horizontal plane, *α*∈[−π/2, π/2], and ω is the angular frequency of the acoustic signal. *ξ*, *η*, and *ζ* are unit vectors along the X-, Y- and Z-axis in the Cartesian coordinate system, respectively. The plane’s wave-sound pressure can be expressed as follows:

(32) p(r,t)=ρcv(r,t)

The velocity components of the three particles are used to describe the sound field as follows:

(33) $$\{\begin{array}{c} \nu_{x}\left(r,t\right)=\frac{p\left(r,t\right)}{\rho c}\cos\theta \cos\alpha \\ \nu_{y}\left(r,t\right)=\frac{p\left(r,t\right)}{\rho c}\sin\theta \cos\alpha \\ \nu_{z}\left(r,t\right)=\frac{p\left(r,t\right)}{\rho c}\sin\alpha \end{array}$$

(34) $$\{\begin{array}{c} \theta =\arctan\left(v_{y}/v_{x}\right)\\ \alpha =\arctan\left(v_{z}/\sqrt{v_{x}^{2}+v_{y}^{2}}\right) \end{array}$$

From Equations (33) and (34), it can be concluded that if the vibration velocity component of the particle can be measured, then the azimuth angle *θ* and elevation angle *α* on the horizontal plane can be obtained. In the far-field approximation, *α* = 0. By measuring the movement direction of the underwater acoustic particle, the pointing map of the vector of the underwater acoustic sensor in the X direction is two tangent space spheres symmetrical to the X-O-Y plane, and it shows “8”-shaped directivity in the X-O-Z plane. The hydrophone can detect the vector information propagated by the target sound source in the sound field by measuring the movement direction of the underwater acoustic particle.

## 5. Simulation Analysis

**Figure 6:**
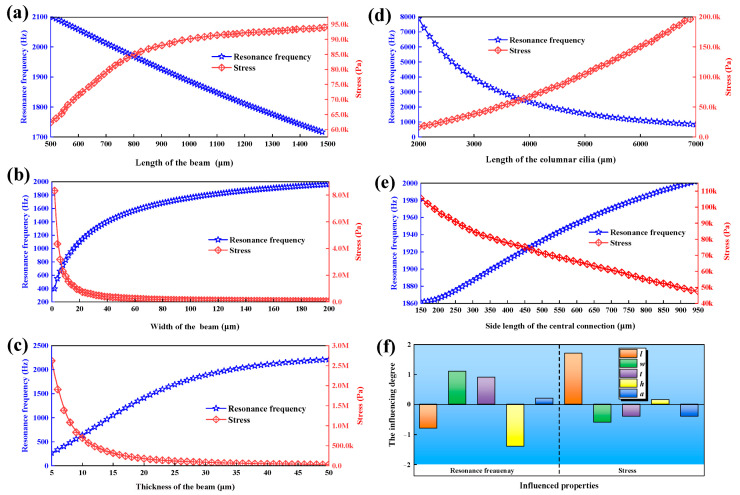
The stress- and resonance-frequency-variation curves of the corresponding dimensional parameters. (**a**–**e**) The resonance frequency and stress-variation curves of the corresponding dimensional parameters, and (**f**) the influencing degrees of the stress and resonance frequency on the microstructural parameters.

MEMS technology uses a semiconductor manufacturing process that enables the production of MEMS sensors on a large-scale with easy integration and compatibility with standard semiconductor manufacturing processes. It enables the integration of multiple sensor units onto a single chip. The multi-unit hydrophone has a square shape and consists of *N* × *N* units, following the structure of a one-unit hydrophone. This simplifies the fabrication process, allowing for easy production of the multi-unit hydrophone using the existing one-unit hydrophone manufacturing process with ease. The FPVH structure consists of four OPVHs with identical structures, so the simulation analysis employs the structure parameters of the one-unit hydrophone. The structure parameters of the one-unit hydrophone are optimized using the COMSOL Multiphysics 6.1 software. When determining the size of the microstructure, two critical factors must be considered. Firstly, the sensitivity is directly proportional to the stress on the beam; if the stress is higher, then the sensitivity is greater, and vice versa. Secondly, improving the first-order resonance frequency of microstructures maximizes the working-frequency range of the hydrophone. To determine the specific size of microstructure, the maximum sensitivity of the microstructure is simulated as an objective function. The constraints are implemented to obtain the optimum solution using COMSOL Multiphysics 6.1 software. During the simulation, it is possible to estimate the structural parameters by observing the changes in stress and sensitivity of the microstructure. Considering the manufacturing process and sensor chip parameters, the design and optimization of the microstructure are performed by using the following parameter ranges: The resonance frequency and stress-variation curves of the corresponding dimensional parameters 0.5 mm < *l* < 1.5 mm, 0 mm < *b* < 0.2 mm, 0.05 mm < *t* < 0.5 mm, 2 mm < *h* < 7 mm, and 0.15 mm < *a* < 0.95 mm. The stress and resonance-frequency-variation curves of the corresponding dimensional parameters are shown in Figure 6a–e. The sensitivity of the microstructure is inversely proportional to the stress on the cantilever beam. This means that, as stress increases, sensitivity also increases, and vice versa. The sensitivity is also proportional to *l* and *h*, and it is inversely proportional to *b*, *t*, and *a*. The trends of stress and structural parameters vary inversely to sensitivity. In summary, the greater the maximum stress on the cantilever beam, the higher the sensitivity of the sensor receiving. The higher the resonance frequency of the suspension beam, the narrower the working bandwidth of the sensor. The sensor’s receiving sensitivity and working-frequency band are mutually restricted, which is consistent with the theoretical result in Section 2.3. Figure 6f shows the dependency level of the stress and resonance frequency on the structural parameters. The bar chart shows that the resonant frequency is more dependent on *h* but is less dependent on *a.* The stress is more dependent on *l* but is less dependent on *a.* The parameter *l* has the greatest positive influence on the maximum stress and the greatest negative influence on resonant frequency on the cantilever beams, while the parameter *h* is totally the opposite. Therefore, selecting the appropriate combination of different parameter sizes is crucial. The simulation optimization progress has two design purposes: (1) FPVH has a better sensitivity than OPVH; and (2) FPVH retains its bandwidth at more than 1000 Hz as much as possible. Based on above the simulation results, MEMS technology, and current processing conditions, the parameters used to optimize the multi-unit microstructure are shown in Table 1.

**Figure 7:**
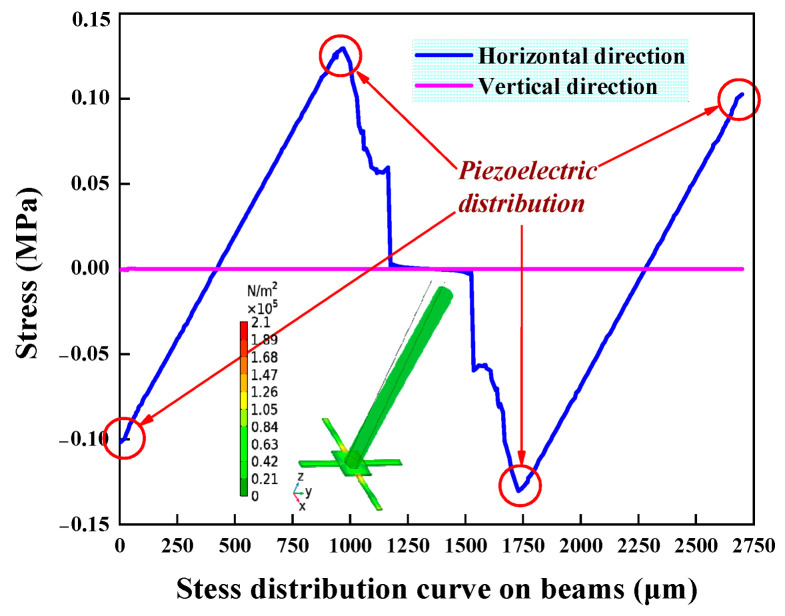
Simulation results of the stress nephogram and stress-distribution curve.

To determine the deformation and stress distribution of the biomimetic cilia microstructure, coupling analysis of the OPVH was performed using COMSOL Multiphysics 6.1 software. An acceleration of 1 *g* was applied along the horizontal direction of the cilia. Figure 7 shows the stress nephogram and stress-distribution curve of the OPVH. The impact of acceleration on the cilium of the hydrophone results in the cantilever beam bending and deforming due to the swing of rigid cilia. The maximum stress of the microstructure is 0.14 MP, which is below the fracture strength of Si (75 MPa). Consistent with our expectations, the stress concentration regions of the beam are distributed at the beam–mass interface and near the support frame. These areas correspond to the optimum distribution of the PZT, which results in the achievement of the maximum stress and voltage output. The symmetry of the microstructure makes the vertical direction equivalent to the horizontal direction. Furthermore, when the cilia apply acceleration in the horizontal direction, the stress generated in the vertical direction is almost negligible. This demonstrates the directional ability of OPVH.

**Figure 8:**
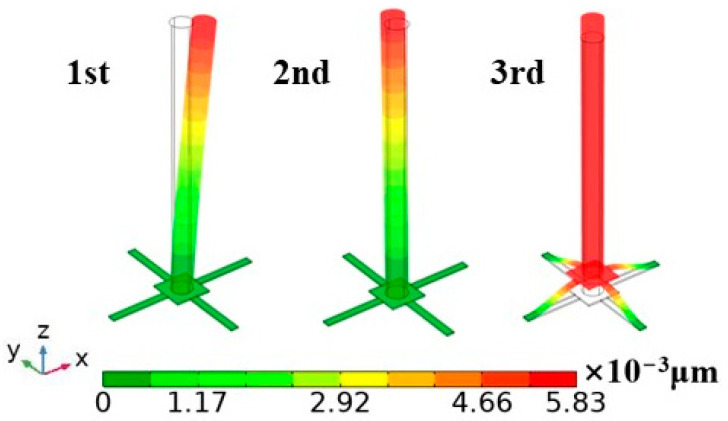
Simulation result of modes.

The modal shapes of the OPVH are shown in Figure 8. The 1st- and 2nd-modal rotations of the cilia occur around the Y-axis and X-axis, respectively. The third-order modal cilia and their supporting block move along the Z-axis. The 3rd-order modal resonance frequencies of the OPVH are 1880.8 Hz, 1881.5 Hz, and 9923 Hz, respectively. It is noteworthy that the 1st-order and 2nd-order resonance frequencies of the OPVH are equal and are lower than the third-order modal. This is owing to the fact that the additional mass of the 3rd-order is greater than that of the first two orders. Therefore, the sound reception capability of the entire structure can be guaranteed in the underwater environment for diverse sound frequencies.

## 6. Fabrication and Experiment

### 6.1. Fabrication

**Figure 9:**
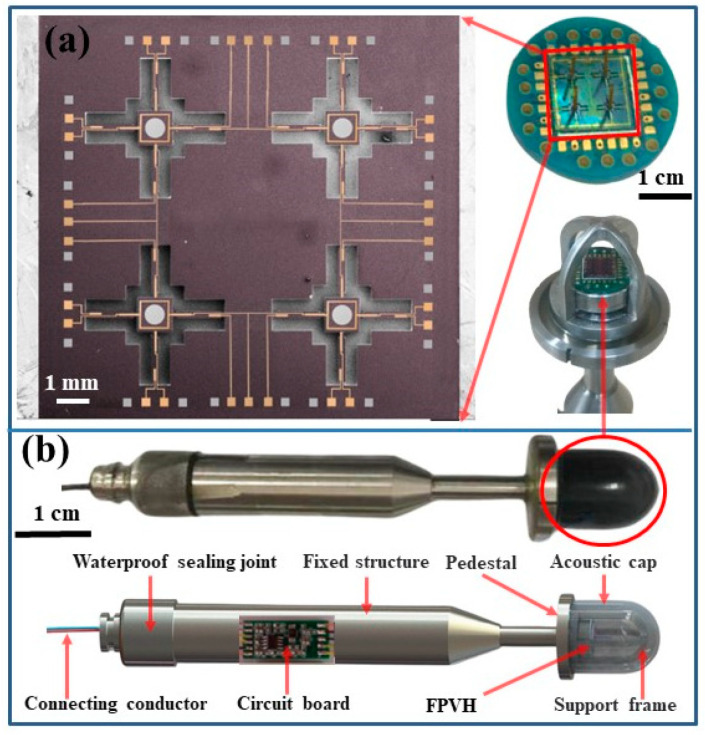
The MEMS array microstructure and the prototype FPVH. (**a**) SEM images and the physical photograph of microstructure, and (**b**) the prototype FPVH.

The four-unit array microstructure was fabricated on the silicon substrate using MEMS technology. The 2 × 2 array units were chosen for ease of implementation with the minimum square array. The critical processes include oxidation, Sol-gel technology, annealing, sputtering, photo-etching, Plasma Enhanced Chemical Vapor Deposition (PECVD), Reactive Ion Etching (RIE), and Ion Beam Etching (IBE). The Scanning Electron Microscope (SEM) images of the fabricated array microstructure are basically consistent in design size, as shown in Figure 9a. The fabrication of the piezoelectric film region is one of the crucial processes. The output voltage of each cantilever beam can only remain constant with the identical distribution of piezoelectric films. Sol-gel technology was utilized to prepare PZT (PbZr_0.53_Ti_0.47_O_3_) [24] on a Si substrate to achieve piezoelectric film homogeneity and adhesion. This technology is compatible with advanced photolithography and ion etching. A good consistency of all PZT piezoelectric units on the crosshead is observed with correct regulation of IBE energy, annealing time and temperature. The array microstructure was stuck to the PCB using a UV-curable adhesive at room temperature. Finally, the plastic column cilia were attached to the central inertial unit with UV-curable adhesive using a dedicated integration platform at room temperature and pressure. The dedicated integration platform mainly includes the monitor, optical CCD system, 3D clamping sleeve device, chip-fixing fixture, and auxiliary tools. Figure 9a shows the physical photograph of the FPVH, which is attached to the plastic column and packaged. Hydrophones are utilized for detecting underwater targets, but their core components lack waterproof capabilities. The complex underwater environment presents many uncertainties, including high-salinity-seawater corrosion, aquatic-organism attacks, and turbulence generated by irregular water movement, all of which may affect hydrophone operation [25]. Therefore, waterproof packaging is a crucial manufacturing process for hydrophones. The most commonly used packaging structure for hydrophones is currently an acoustically transparent cap. This paper adopts a packaging method that combines an acoustically transparent cap and an internal support structure, using ultra-thin, high-transmission and highly elastic polyurethane as the waterproof acoustic transparent cap material, with stainless steel for the internal lining. To ensure proper sound transmission, it is necessary to inject insulating medium oil into the polyurethane sound cap. Silicone oil is commonly used as the sound propagation medium due to its similar density, wave speed, and characteristic impedance to water. The oil should be injected into the sound penetration cap through the oil inlet hole of the encapsulation shell, and any air in the sound penetration cap should be discharged through the oil outlet hole. After filling the sound penetration cap with silicone oil and confirming the absence of bubbles, the oil injection hole should be closed with sealant. Bubbles in the sound penetration cap can cause changes in the sensitivity of the underwater acoustic sensor with changes in hydrostatic pressure [25]. Therefore, when injecting silicone oil into the sound through cap, several aspects should be considered: (1) Before injecting silicone oil into the sound transmission cap, preheat and evacuate the silicone oil to remove any bubbles. (2) Once cooled, inject the silicone oil through the oil inlet hole and discharge any bubbles inside the sound transmission cap through the oil outlet hole. (3) Immerse the underwater acoustic sensor, filled with silicone oil, into a large beaker also filled with silicone oil. Ensure that the oil inlet and outlet holes on the tube shell are fully immersed in silicone oil. (4) Vacuum the probe again to ensure that any bubbles inside the sound transmission cap are removed. (5) Once complete, seal the oil-filling hole with sealant. To output the available electrical signals, a low-noise pre-amplifier circuit is also located inside the support structure. The prototype of the FPVH is shown in Figure 9b, and it is mainly composed of a connecting conductor, waterproof sealing joint, circuit board, fixed structure, pedestal, support frame, and acoustic cap.

### 6.2. Experimental Equipment and Characteristics

**Figure 10:**
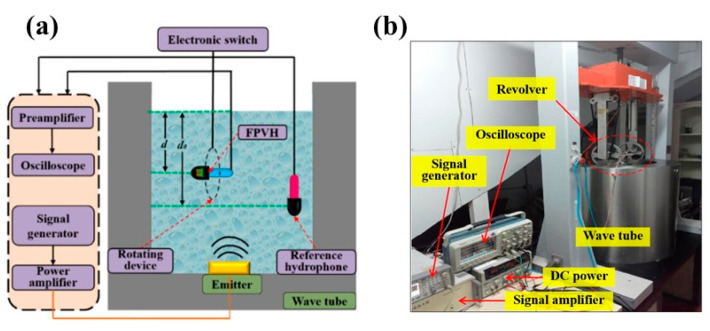
Wave tube calibration device. (**a**) Schematic diagram of wave tube calibration device and (**b**) calibration experiment site.

The vector hydrophone is utilized for detecting signals from underwater targets, and it is crucial to conduct testing and analysis on two key characteristics: receiving sensitivity and directionality. Calibration experiments (North University of China, Taiyuan, China) involve using a standing wave tube to measure the sensitivity and directional response of hydrophones relative to the frequency of underwater acoustic signals [26]. As shown in Figure 10a, the calibration device consists of a wave tube, signal generator, signal transducer, power amplifier, voltage regulator, digital oscilloscope, testing system chassis, and electronic switch device. The wave tube can be used to ensure that the hydrophone is in the plane-wave field in which both the FPVH hydrophone and the standard hydrophone are placed. To determine the receiving sensitivity and directional response of the FPVH, the output signals of the FPVH are compared to those of a standard hydrophone. The sensitivity calibration test was conducted by fixing the sound source at the bottom of the wave tube and emitting sound waves upward under the excitation of the power amplifier to form a free sound field [27]. The standard hydrophone is positioned upside-down on a lifting table to ensure that its head is vertically oriented, effectively receiving the sound source signal emitted by the signal transducer.

The testing site of the FPVH is shown in Figure 10b. After turning on the power, it is necessary to wait for 15 min to ensure the stable operation of the electrical part of the system. The FPVH is mounted on a mechanical rotating device with a bearing-like structure and four orthogonally distributed fixed ends. The signal generator has been configured to produce a sinusoidal wave with a peak-to-peak voltage of 1 V, amplified by 20 dB, with a step increments of 1/3 octave, and a sampling frequency range from 20 Hz~2000 Hz. The control console for the lift is set up to position the standard hydrophone and the FPVH at distances of *d*_0_ and *d* from the surface of the water, respectively. The corresponding sound pressure *p*_0_ can be obtained by measuring the open-circuit voltage *e_0_* of the standard hydrophone. Likewise, measuring the FPVH no-load output voltage and the sound-pressure information at the standard hydrophone location and calculating the doubled data provides the FPVH sensitivity value for each frequency point. According to the definition of hydrophone output in free field, the sensitivity of the standard hydrophone and FPVH under test are given by the Equations (35) and (36), respectively [28]:

(35) $$M_{0}=e_{0}/p_{0}$$

(36) $$M_{x}={e_{x}/p}_{x}$$

where *M*_0_ and *M_x_* are the sensitivities of the standard hydrophone and the FPV, respectively. In this work, the value of *M*_0_ is −180 dB. *e*_0_ and *e_x_* are the standard and FPVH hydrophone output voltages, respectively. The sound pressure complies with *p*∝sin*(kd)* in the wave tube, where *p*_0_ and *p_x_* represent the underwater depths of the FPVH and the standard hydrophone, respectively. Therefore, *M_x_* can be expressed as

(37) $$M_{x}=20\mathrm{lg}(\frac{e_{x}}{e_{0}}\frac{\sin kd}{\cos kd_{0}})+M_{0}$$

where *d* and *d*_0_ represent the underwater depth of the FPVH and the standard hydrophone, respectively, and the wave number is *k*. In the calibration process, the hydrophones were placed in the same horizontal plane (*d = d*_0_).

**Figure 11:**
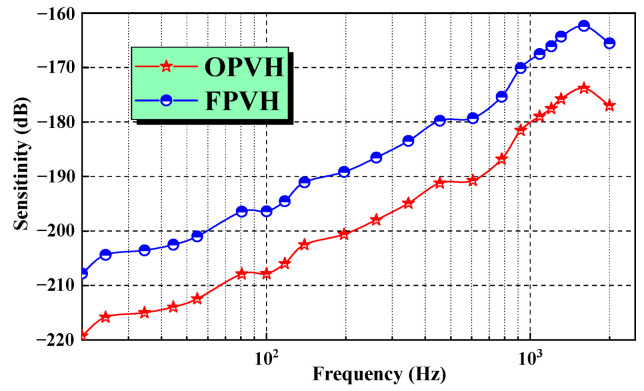
Sensitivity-contrast curves of OPVH and FPVH.

The sensitivity-contrast curves of the OPVH and FPVH are shown in Figure 11. The sensitivity of the OPVH and FPVH were −179.13 dB@1000 Hz and −167.93 dB@1000 Hz (0 dB = 1 V/μPa), respectively. Compared with the one-unit hydrophone, the sensitivity of four-unit array hydrophone is improved by about 12 dB at 1000 Hz. It has been proved that the sensitivity of piezoelectric hydrophone is better than that of the piezoresistive hydrophone, and the characteristics of FPVH are better than those of the OPVH. However, the fabrication process of the FPVH is not more complex. The first resonance peak appeared at 1800 Hz, which suggests that the working bandwidth of the FPVH is approximately 20 Hz~1200 Hz, as the upper limit of the working frequency is two-thirds of the resonant frequency.

The directivity was calibrated in the same wave tube calibration device, using the same suspension method described in above section, with elastic suspension on a mechanical rotating-rod structure. The standing wave barrel excites a standing wave signal via the bottom transmitting transducer, which is perpendicular to its bottom plane. Therefore, the FPVH is rotated synchronously by a rotating mechanical rod to simulate the reception of sound signals from a 0° to 360° direction while the hydrophone remains in a fixed position. During the directional calibration of the FPVH, a continuous standing wave signal is emitted from the transmitting transducer at any frequency point, and the output voltage of the FPVH is recorded at different angles by rotating the mechanical rotating bar in 5° steps. The recorded data is normalized according to Equation (38) to obtain the directivity [29].

(38) $$L=20\log D(\theta )=20\log(\frac{e_{\theta }}{e_{\max}})$$

where *e_θ_* is the output voltage of the FPVH on rotation, while *e_max_* is the maximum output at this time.

**Figure 12:**
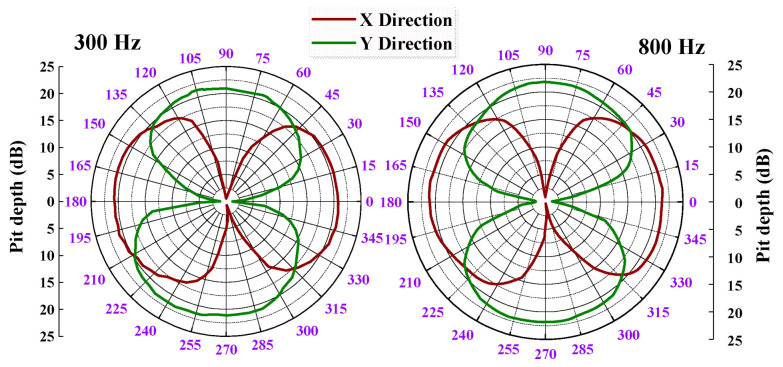
Directivity patterns of the FPVH at 300 Hz and 800 Hz.

The directivity pattern of the FPVH can be obtained by plotting the normalized measurement data in polar coordinates at a given frequency. Figure 12 shows the directivity patterns at 300 and 800 Hz. The results show that the patterns have a good orthogonality between the X and Y directions, and a good cosine curve with an 8 shape. The high frequency range is smoother and more symmetrical than the low-frequency range. Axial sensitivity has a maximum variation of 0.13 dB, and the depth of the troughs exceeds 20 dB, indicating excellent directivity. However, the directivity diagram of the vector hydrophone is not an ideal way to meet the requirement for an excellent 8 shape. The non-vertical adhesion of cilia can cause a slight asymmetry in the directivity pattern.

Table 2 shows a comparison of the performance of the developed MEMS array hydrophone with previous versions. The FPVH has proven to be a competitive high-performance device in terms of sensitivity and working bandwidth when compared to previous MEMS array hydrophones. In this work, the FPVH shows higher sensitivity than our previous work [9], with the sensitivity of the four-unit array hydrophone improved by about 12 dB at 1000 Hz. The sensitivity of piezoelectric array hydrophones is approximately 10 dB higher than that of piezoresistive array hydrophones with this cilia structure. Additionally, the working bandwidth of piezoelectric array hydrophones has been broadened. The AIN-on-SOI platform array hydrophones have an obvious lower sensitivity and narrower operating bandwidth. The honeycomb AlN- and ScAlN-based array hydrophones have a wider operating bandwidth, which makes them less resistant to interference. However, no systematic study has been conducted on directivity.

## 7. Conclusions

By studying the mechanism of fish cilia, an FPVH was designed and manufactured to mimic their ability to detect sound in water. The sensitivity of the FPVH reaches up to −167.93 dB at 1000 Hz, which is much higher than that of the OPVH, and the sensitivity of the multi-unit piezoelectric hydrophone is improved by 10.6 dB compared to the piezoresistive hydrophone with the same array number. The FPVH maintains sufficient bandwidth while improving on the sensitivity of the single-unit hydrophone. Additionally, the bandwidth of the FPVH reaches 20 Hz~1200 Hz while exhibiting satisfactory 8-shaped directivity. The results of this work pave a new way for the development of multi-unit piezoelectric vector hydrophones for underwater communications and acoustic detectors.

## Figures and Tables

**Table 1:** The parameters of the multi-unit microstructure.

Structure Parameters	Parameter Value (μm)
Length of beam (*l*)	1000
width of beam (*b*)	130
thickness of beam (*t*)	35
side length of central inertial unit (2*a*)	700
height of cilium (*h*)	4500
diameter of cilium (*D*)	400
side length of one-units (*N* × *N* units)	5000 × N

**Table 2:** Characteristics comparison of MEMS array hydrophones.

Hydrophones	Material	Sensitivity	Bandwidth	Directivity	Ref.	Technology
FPVH	PZT	−167.93 dB@1000 Hz	20 Hz~1200 Hz	“8”	This work	piezoelectric
OPVH	PZT	−179.13 dB@1000 Hz	20 Hz~1200 Hz	“8”	[9]	piezoelectric
FCVH	resistance	−177.14 dB@1000 Hz	20 Hz~1000 Hz	“8”	[13]	piezoresistive
AIN-on-SOI	AIN	−182.5 dB (re: 1 V/μPa)	10 Hz~100 Hz	“omni”	[15]	piezoelectric
Honeycomb	AlN	−178 dB (re: 1 V/μPa)	10 Hz~50 kHz	/	[16]	piezoelectric
Honeycomb	ScAlN	−164.5 dB (re: 1 V/μPa)	10 Hz~50 kHz	/	[17]	piezoelectric

## Data Availability

Data are contained within the article.
